# Repeatability and Reproducibility of Retinal Neuronal and Axonal Measures on Spectral-Domain Optical Coherence Tomography in Patients with Cognitive Impairment

**DOI:** 10.3389/fneur.2017.00359

**Published:** 2017-08-15

**Authors:** Edwin Hong-Teck Loh, Yi-Ting Ong, Narayanaswamy Venketasubramanian, Saima Hilal, Naing Thet, Tien Yin Wong, Christopher P. L. Chen, Carol Yim-lui Cheung

**Affiliations:** ^1^Singapore Eye Research Institute, Singapore National Eye Centre, Singapore, Singapore; ^2^Duke-NUS Medical School, National University of Singapore, Singapore, Singapore; ^3^Memory Aging and Cognition Centre, National University Health System, Singapore, Singapore; ^4^Raffles Neuroscience Centre, Raffles Hospital, Singapore, Singapore; ^5^Department of Ophthalmology, Yong Loo Lin School of Medicine, National University of Singapore, Singapore, Singapore; ^6^Department of Pharmacology, National University of Singapore, Singapore, Singapore; ^7^Department of Ophthalmology and Visual Sciences, The Chinese University of Hong Kong, Hong Kong, Hong Kong

**Keywords:** retina, reproducibility, repeatability, mild cognitive impairment, dementia, optical coherence tomography

## Abstract

**Background:**

With increasing interest in determining if measurement of retinal neuronal structure with spectral-domain optical coherence tomography (SD-OCT) is useful in accessing neurodegenerative process in cognitive decline and development of dementia, it is important to evaluate whether the SD-OCT measurements are repeatable and reproducible in these patients.

**Methods:**

This is a retrospective cohort study. Patients with Alzheimer’s disease (AD) or mild cognitive impairment (MCI) with no change in global clinical dementia rating (CDR) score at 1-year follow-up were eligible to be included. Ganglion cell-inner plexiform layer (GC-IPL) and retinal nerve fiber layer (RNFL) parameters were measured with SD-OCT at baseline, 6-month, and 1-year follow-up visits. At baseline, SD-OCT scans were repeated to access intra-visit repeatability of the SD-OCT measurement. SD-OCT measurement over three visits was used to access inter-visit reproducibility. We calculated intraclass correlation coefficients (ICC) and coefficients of variation (CoVs).

**Results:**

We included 32 patients with stable AD and 29 patients with stable MCI in the final analysis. For GC-IPL measures, the average intra-visit ICC was 0.969 (range: 0.948–0.985), and CoV was 1.81% (range: 1.14–2.40); while the average inter-visit ICC was 0.968 (0.941–0.985), and CoV was 1.91% (range: 1.24–2.32). The average ICC and CoV of intra-visit RNFL measured were 0.965 (range: 0.937–0.986) and 2.32% (range: 1.34–2.90%), respectively. The average ICC and CoV of inter-visit RNFL measures were 0.927 (range: 0.845–0.961) and 3.83% (range: 2.71–5.25%), respectively.

**Conclusion:**

Both GC-IPL and RNFL measurements had good intra-visit repeatability and inter-visit reproducibility over 1 year in elderly patients with no decline in cognitive function, suggesting that SD-OCT is a reliable tool to assess neurodegenerative process over time.

## Introduction

The retina shares similarities with cerebral neurons in development, physiology, and anatomy (1–3). Retinal optical coherence tomography (OCT), a non-invasive *in vivo* optical biopsy of the retina, has enabled remarkable advances in assessing retinal ganglion cell (RGC) axons by quantifying peripapillary retinal nerve fiber layer (RNFL) damage, particularly in the field of glaucoma (4). OCT has also been used to study neurodegenerative diseases such as dementia (5). Current clinical studies have shown that RNFL measured by OCT exhibits thinning in patients with mild cognitive impairment (MCI) and Alzheimer’s disease (AD) (6–16). With spectral-domain OCT (SD-OCT), a greater degree of resolution and scan speed is now available to allow more detailed RGC analysis at the macular region including measurement of the ganglion cell layer that is composed of cell bodies, and the inner plexiform layer that contains the RGC dendrites (17, 18). It has been shown that macular ganglion cell-inner plexiform layer (GC-IPL) neuronal loss is more strongly related to MCI, compared with RNFL axonal loss, suggesting that GC-IPL thickness is a more sensitive marker than RNFL thickness for assessing neurodegenerative pathology in MCI or early AD (11).

Numerous studies have demonstrated good reproducibility and repeatability of using SD-OCT to measure RNFL and GC-IPL thicknesses in normal, glaucoma, retinal diseases as well as hypertensive eyes (14, 19–25). However, data on the repeatability and reproducibility in patients with cognitive dysfunction and dementia are lacking. One study reported the repeatability of OCT in measurement of retinal and RNFL thicknesses in 75 patients with AD, with satisfactory results [mean coefficient of variation (CoV) of 4.78% and ICC of >0.905] (26), though the study only involved intra-visit analysis but no inter-visit analysis and no assessment of GC-IPL thickness.

It is crucial to ascertain both the intra-visit repeatability and inter-visit reproducibility of OCT measurements in patients with cognitive dysfunction, to ensure changes detected are indeed due to pathological neurodegeneration in patients with progressive cognitive impairment, rather than machine error or natural regression due to age. In this study, we assessed intra-visit repeatability and inter-visit reproducibility of macular GC-IPL and peripapillary RNFL measurement with SD-OCT in patients with cognitive impairment.

## Materials and Methods

### Study Population

All patients were recruited from memory clinics at the National University Hospital, Singapore, as part of an ongoing prospective study of cognitive progression. Patients were eligible to be recruited if they were diagnosed with dementia syndrome (Alzheimer’s type) in accordance to the Diagnostic and Statistical Manual of Mental Disorders, 4th edition (DSM-IV) or MCI (Petersen’s criteria) (27). Subjects underwent cognitive assessment by neuropsychologists at baseline and 1-year follow-up. Assessment for severity of cognitive impairment included clinical dementia rating (CDR) scale. In the current analysis, only subjects determined to be stable (no change in global CDR score) at 1-year follow-up, with no history of glaucoma (28, 29), and completed OCT scans at all visits were included.

Written informed consent was obtained from each participant or his or her primary caregiver; the study conducted adhered to the Declaration of Helsinki. Ethics approval was obtained from National Healthcare Group Pte Ltd Domain Specific Review Board.

### OCT Imaging

After pupil dilation using tropicamide 1.0% and phenylephrine hydrochloride 2.5%, a spectral-domain OCT (Cirrus HD-OCT, software version 6.0.2, Carl Zeiss Meditec, Dublin, CA, USA) was used to obtain scans using the cube 200 × 200 and optic nerve head cube 200 × 200 scan protocols, respectively, in each eye in a darkened room. Eye-tracking function was not available in this software version. Before imaging, our trained technicians explained the details of imaging procedures and clearly instructed the subjects on how to look at the fixation target without blinking during imaging. At least two attempts per scan per eye were taken at each visit. At baseline visit, two consecutive scans were taken for each scan protocol and each eye for assessment of intra-visit repeatability. Scans were repeated at 6-month and 1-year follow-up visits for assessment of reproducibility.

After imaging, all the OCT data were sent to our reading center and reviewed by our trained graders. Assessment of each scan was made by two certified trained graders at the reading center. Adjudication was provided by retinal specialists if needed. The graders checked the quality of scan, presence of pathology, and segmentation of GC-IPL and RNFL. GC-IPL thicknesses were detected and measured automatically from a 14.13 mm^2^ elliptical annulus area centered on the fovea, from macular scans. Peripapillary RNFL thicknesses were measured automatically from a calculation circle of diameter 3.46 mm centered on the optic disk, from optic nerve head scans. An algorithm error was defined as incorrect segmentation of GC-IPL and RNFL. Scans were excluded from analysis if signal strength was less than 5, or when there was retinal pathology affecting GC-IPL or RNFL segmentation (such as epiretinal membrane, macula edema, vitreomacular traction, age-related macular degeneration, etc.), or when there was incorrect segmentation of GC-IPL or RNFL. An APOSTLE checklist for our OCT imaging was included in the Appendix in the Supplementary Material.

### Statistical Analysis

Intraclass correlation coefficients (ICC) and CoV were calculated for each parameter from each SD-OCT scan with acceptable quality for analysis. Parameters assessed include macular GC-IPL (average, superotemporal, superior, superonasal, inferonasal, inferior, and inferotemporal) and peripapillary RNFL (average, temporal, superior, nasal, and inferior) thicknesses. Intra-visit repeatability was assessed using measurements from two consecutive scans performed at the baseline visit, while inter-visit reproducibility was assessed using measurements over all three visits (baseline, 6-month, and 1-year) follow-ups.

In addition, Bland–Altman plots comparing selected parameters such as GC-IPL and peripapillary RNFL average thicknesses from baseline and 1-year follow-up were used to assess for bias.

We also performed a subgroup analysis of subjects with AD and MCI, respectively, to assess if the reproducibility and repeatability was poorer in patients with either AD or MCI.

## Results

**Figure 1:**
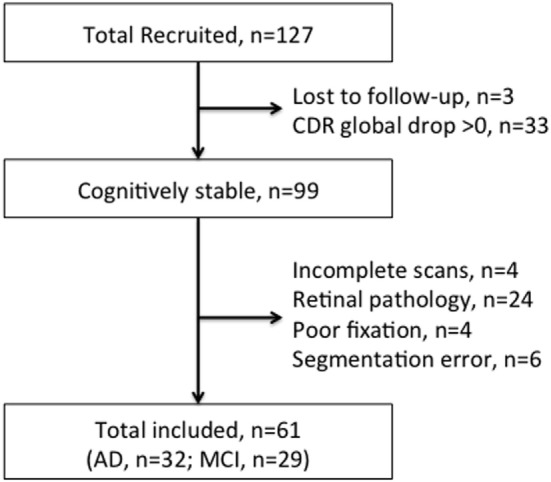
Flowchart of Participants for the overall assessment.

A total of 61 subjects were eligible to be included in the study—32 with AD and 29 with MCI. Figure 1 shows the flowchart of the participants included in the study. Excluded scans in this flowchart reflect subject scans that were unable to be used across all three visits. Appendices 2 and 3 in Supplementary Material show the breakdown of scans excluded from the study by eyes and the main reasons for exclusion, respectively.

Table 1 presents the distribution of subject characteristics at baseline. These subjects had SD-OCT scans with acceptable quality for either GC-IPL or RNFL (or both) measurements. For GC-IPL measures, within the 61 eligible patients, a total of 70 eyes were suitable for intra-visit analysis (37 right and 33 left), while 52 eyes had data over all three visits for inter-visit analysis (26 right and 26 left). For peripapillary RNFL measures, a total of 77 eyes were suitable for intra-visit analysis (35 right and 42 left), while 69 eyes had data over all three visits for inter-visit analysis (37 right and 32 left).

**Table 1:** Baseline characteristics of study subjects.

Baseline characteristics	All subjects (*n* = 61)	Alzheimer’s disease (*n* = 32)	Mild cognitive impairment (*n* = 29)
Age, mean (SD)	73.1 (8.3)	76.3 (5.5)	69.7 (9.5)
Male, *n* (%)	28 (45.9)	13 (40.6)	15 (51.7)
Chinese ethnicity, *n* (%)	45 (73.8)	25 (78.1)	20 (69.0)
Hypertension, *n* (%)	42 (68.9)	27 (84.4)	15 (51.7)
Diabetes, *n* (%)	23 (37.7)	12 (37.5)	11 (37.9)
Hypercholesterolemia, *n* (%)	45 (73.8)	22 (68.8)	23 (79.3)
Clinical dementia rating (CDR) global = 0.5, *n* (%)	29 (47.5)	0 (0.0)	29 (100)
CDR global = 1, *n* (%)	25 (41.0)	25 (78.1)	0 (0.0)
CDR global = 2, *n* (%)	7 (11.5)	7 (21.9)	0 (0.0)

Table 2 shows that the means and SD of parameters from both ONH and macula scans remained similar and were not significantly different across all three visits.

**Table 2:** Distributions of macular ganglion cell-inner plexiform layer (GC-IPL) and peripapillary retinal nerve fiber layer (RNFL) thicknesses.

Mean (SD)	Baseline	6-Month follow-up	1-Year follow-up	*P*
**Macular GC-IPL parameters (μm)**				
GC-IPL average thickness	77.44 (9.56)	77.37 (9.93)	77.13 (9.19)	0.985
GC-IPL minimum thickness	72.54 (12.46)	72.38 (12.67)	72.13 (12.42)	0.986
GC-IPL temporal superior	77.04 (10.78)	76.79 (11.13)	76.58 (9.71)	0.976
GC-IPL superior	78.42 (8.89)	78.17 (9.71)	77.75 (8.94)	0.932
GC-IPL nasal superior	79.37 (10.12)	79.15 (10.14)	79.10 (9.80)	0.990
GC-IPL nasal inferior	76.85 (10.23)	76.77 (10.17)	77.10 (10.20)	0.985
GC-IPL inferior	75.42 (10.75)	75.52 (10.87)	75.31 (10.11)	0.995
GC-IPL temporal inferior	77.38 (10.55)	77.48 (11.26)	76.67 (10.28)	0.915
**Peripapillary RNFL parameters (μm)**				
RNFL average thickness	90.77 (12.48)	90.75 (12.74)	89.54 (12.94)	0.810
RNFL temporal quadrant	65.87 (12.57)	66.19 (13.49)	65.88 (13.16)	0.987
RNFL superior quadrant	110.77 (18.65)	110.20 (17.62)	108.45 (18.17)	0.737
RNFL nasal quadrant	69.06 (10.75)	69.36 (10.13)	68.17 (10.76)	0.789
RNFL inferior quadrant	117.54 (24.38)	117.23 (24.80)	115.54 (24.78)	0.877

Intra-visit repeatability and inter-visit reproducibility of macular GC-IPL thicknesses are shown in Table 3. For macular GC-IPL thicknesses, the range of intra-visit ICC was 0.948–0.985, and the range of CoV was 1.14–2.40%, while the range of inter-visit ICC was 0.941–0.985, and the range of CoV was 1.24–2.32%.

**Table 3:** Intra-visit repeatability and inter-visit reproducibility of macular ganglion cell-inner plexiform layer (GC-IPL) measurement.

Parameters	Coefficient of variation (%)	ICC
**Intra-visit repeatability**		
GC-IPL average thickness	1.14	0.985 (0.976–0.991)
GC-IPL minimum thickness	2.12	0.962 (0.939–0.976)
GC-IPL superotemporal	1.49	0.983 (0.973–0.989)
GC-IPL superior	1.96	0.962 (0.940–0.976)
GC-IPL superonasal	1.51	0.974 (0.959–0.984)
GC-IPL inferonasal	2.33	0.948 (0.918–0.967)
GC-IPL inferior	2.40	0.954 (0.928–0.971)
GC-IPL inferotemporal	1.56	0.983 (0.972–0.989)
**Inter-visit reproducibility**		
GC-IPL average thickness	1.24	0.985 (0.977–0.991)
GC-IPL minimum thickness	1.79	0.983 (0.974–0.990)
GC-IPL superotemporal	1.96	0.960 (0.938–0.976)
GC-IPL superior	2.32	0.941 (0.909–0.964)
GC-IPL superonasal	1.74	0.977 (0.965–0.986)
GC-IPL inferonasal	2.12	0.960 (0.937–0.975)
GC-IPL inferior	2.25	0.966 (0.946–0.979)
GC-IPL inferotemporal	1.86	0.975 (0.960–0.985)

Intra-visit repeatability and inter-visit reproducibility of peripapillary RNFL thicknesses are shown in Table 4. For peripapillary RNFL thicknesses, the range of intra-visit ICC was 0.937–0.986, and the range of CoV was 1.34–2.90%, while the range of inter-visit ICC was 0.845–0.961, and the range of CoV was 2.71–5.25%.

**Table 4:** Intra-visit repeatability and inter-visit reproducibility of peripapillary retinal nerve fiber layer (RNFL) measurement.

Parameters	Coefficient of variation (%)	ICC
**Intra-visit repeatability**		
RNFL average thickness	1.34	0.986 (0.978–0.991)
RNFL temporal quadrant	2.51	0.951 (0.924–0.969)
RNFL superior quadrant	2.40	0.972 (0.956–0.982)
RNFL nasal quadrant	2.90	0.937 (0.904–0.960)
RNFL inferior quadrant	2.48	0.977 (0.964–0.985)
**Inter-visit reproducibility**		
RNFL average thickness	2.71	0.952 (0.928–0.968)
RNFL temporal quadrant	3.38	0.961 (0.942–0.974)
RNFL superior quadrant	3.84	0.922 (0.885–0.948)
RNFL nasal quadrant	5.25	0.845 (0.781–0.896)
RNFL inferior quadrant	3.97	0.955 (0.934–0.971)

**Figure 2:**
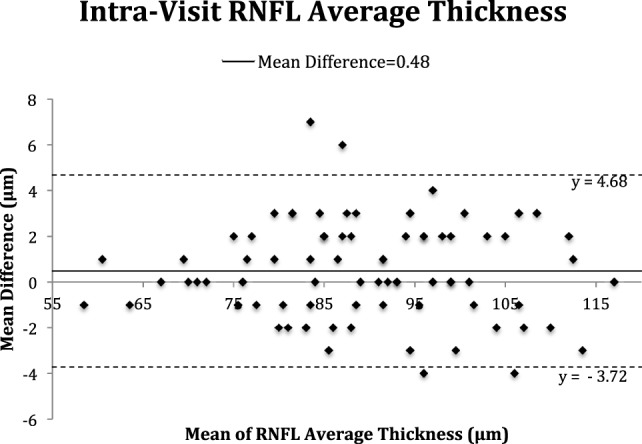
Bland–Altman plot of intra-visit peripapillary retinal nerve fiber layer (RNFL) thickness.

**Figure 3:**
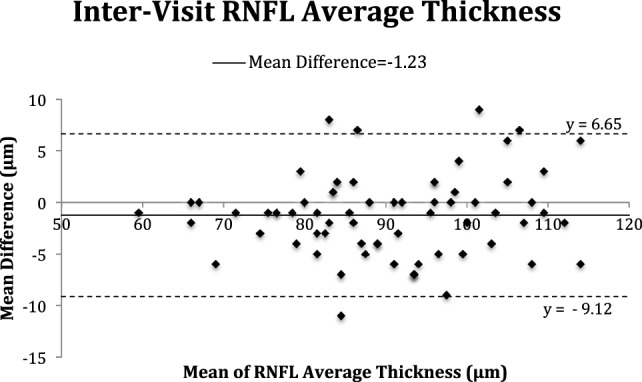
Bland–Altman plot of inter-visit peripapillary retinal nerve fiber layer (RNFL) thickness.

**Figure 4:**
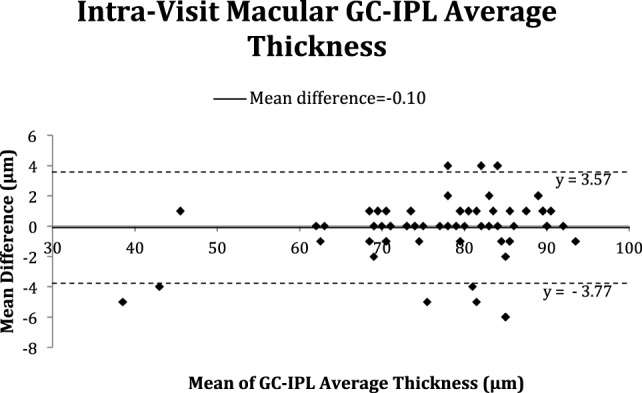
Bland–Altman plot of intra-visit macular ganglion cell-inner plexiform layer (GC-IPL) average thickness.

**Figure 5:**
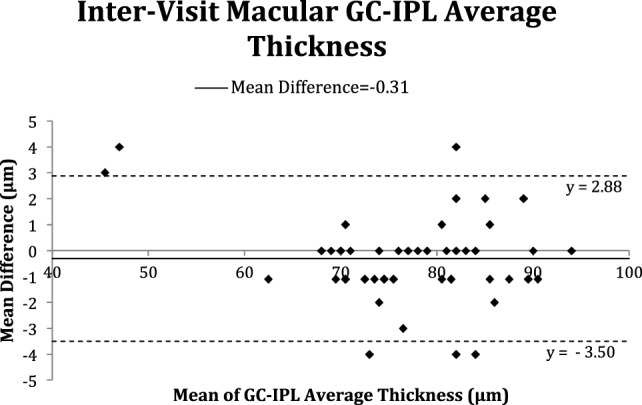
Bland–Altman plot of inter-visit macular ganglion cell-inner plexiform layer (GC-IPL) average thickness.

The Bland–Altman plots (Figures 2–5) obtained for peripapillary RNFL and macular GC-IPL thicknesses intra and inter-visits showed no evidence of bias in intra- and inter-visit mean differences in both RNFL and GC-IPL average thickness measurements.

In the subgroup analysis (Tables S1–S4 in Supplementary Material), we found that the values of CoV and ICC for GC-IPL and RNFL measurements were similar between subjects with AD and MCI, indicating both GC-IPL and RNFL measures had similar reliability performance between AD and MCI groups.

## Discussion

The results obtained from this study highlight that GC-IPL and RNFL measurements obtained using SD-OCT were repeatable and reproducible in cognitively impaired patients with no significant cognitive decline over 1 year.

Optical coherence tomography is a promising and potential tool to assess neurodegenerative diseases such as dementia. Recent clinical studies have shown that thinning of the GC-IPL and RNFL in the retina is present in patients with MCI and AD when compared with normal controls (6–10), suggesting that OCT measurements may be used as a biomarker to assess neuronal loss. However, there was doubt if these changes were due to disease mechanisms or could be attributed to measurement variability or natural progression due to age (30, 31) and other factors unrelated to cognitive neurodegeneration. There was also no available evidence on whether RNFL and GC-IPL continued to degenerate in cognitively impaired patients with relatively stable conditions, if thinning was independent or associated with cognitive performance.

Numerous studies have examined reproducibility and repeatability of using OCT to assess RNFL measurement in normal healthy subjects, glaucoma, retinal diseases, and hypertensive eyes, with ICCs ranging from around 0.90 to 0.99, and CoV ranging from around 0.41 to 2.24% (19–25). Furthermore, one study reported the repeatability of OCT in measurement of retinal and RNFL thicknesses in 75 patients with AD, with satisfactory results (mean CoV of 4.78% and ICC of >0.905) (23). Our study provides additional data showing that both intra-visit repeatability and inter-visit reproducibility was excellent, with inter-visit ICCs ranging from 0.845 to 0.961, and the range of CoV was 2.71–5.25%. In addition, we also extended our study population to those with MCI, with similar repeatable and reproducible OCT measurement.

Furthermore, in studies employing similar imaging techniques on patients with MCI and AD, patients had a reduction of 12.40–20.00 µm in the RNFL average thickness (12), a reduction of 27.10–30.09 µm in the RNFL superior quadrant thickness, and a reduction of 3.42–4.99 µm in average GC-IPL thickness (11) when compared with normal controls. Given the results of our study, which showed a CoV of 2.71% for the inter-visit RNFL average thickness and 1.24% for inter-visit GC-IPL average thickness, the acceptable and expected variability of the measurements for average thickness of RNFL (mean 90.23 µm) and GC-IPL (mean 76.75 µm) are about 2.44 and 0.95 µm, respectively.

Since reduction in both RNFL and GC-IPL thicknesses in both MCI and AD patients was much larger than the expected variation in persons with stable cognitive statuses, it suggests that retinal degeneration observed by these previous studies is likely to be due to disease, rather than measurement variability. However, whether reduction of RNFL and GC-IPL thicknesses in patients is indicative or predictive of future cognitive decline needs to be further explored in prospective studies.

Hence, this study examined and concluded that the repeatability and reproducibility of the data obtained patients with MCI and AD from both intra and inter-visit OCT scans across three visits were excellent. Importantly, this shows that it can accurately and reliably detect retinal changes in patients and these changes could be directly attributed to the patients’ disease process, rather than measurement variability due to OCT. It also provides strong validation data from previous studies showing RNFL and GC-IPL thinning in patients with neurodegenerative diseases.

Strengths of the study include standardized imaging protocols and strict quality control to ensure that OCT scans obtained were of consistent quality and using standardized objective cognitive function assessment tools to monitor cognitive decline in subjects. Furthermore, there was the availability of a SD-OCT for use to collect data from the involved patients and the inclusion of patients with varying degrees of cognitive impairment. Moreover, we also ruled out confounding ocular pathologies to ensure any observed variance is not due to ocular pathology.

However, a limitation of our study was that only patients with good quality scans across all three-study visits were included for analysis. Hence, these results may not be generalizable to non-compliant patients, especially those with severe cognitive impairment. However, strict inclusion criteria were implemented to prevent introducing variability to measurements from factors other than cognitive function or pathological neurodegeneration. Also, the strict criteria ensures that the data collected and analyzed were accurate and in line with our study goals as much as possible. Second, the current OCT model lacked eye-tracking capabilities. This might affect the reliability of the measurements obtained. Another limitation of our study was the elimination of ungradable scans and eyes with retinal pathology. This may underestimate and limit the application of the data to real-world situations. Finally, all images were taken at a single site, and this might have introduced a degree of bias.

In conclusion, OCT is a commonly and widely used diagnostic tool and has aided in the diagnosis and follow-up for many patients worldwide. Its use and role in clinical assessment are ever expanding. This study showed that both RNFL and GC-IPL measurements had good reproducibility over 1 year in elderly patients with no decline in cognitive function. It gives great promise to the ability of OCT to detect changes in the retinal layers that could be directly associated with the disease process in patients with MCI and AD, suggesting that SD-OCT may be useful in uncovering pathological changes in retinal neuronal and axonal layers associated with cognitive decline.

## Ethics Statement

This study was carried out in accordance with the recommendations of “National Healthcare Group Pte Ltd Domain Specific Review Board” with written informed consent from all subjects. All subjects gave written informed consent in accordance with the Declaration of Helsinki. The protocol was approved by the “National Healthcare Group Pte Ltd Domain Specific Review Board.”

## Author Contributions

EH-TL and Y-TO—substantial contributions to the design, acquisition, analysis, and interpretation of data and drafting of the manuscript. NV—substantial contributions to the design, interpretation of data and critical revision for intellectual content. SH and TW—substantial contributions to the acquisition, analysis, and interpretation of data and critical revision for intellectual content. NT—substantial contributions to the analysis, interpretation of data and critical revision for intellectual content. CC—substantial contributions to the design, acquisition, analysis, and interpretation of data and critical revision for intellectual content. CY-lC—substantial contributions to the design, analysis, and interpretation of data and critical revision for intellectual content. All the authors gave final approval of the version to be published and were agreed to be accountable for all aspects of the manuscript in ensuring that questions related to the accuracy or integrity of any part of the work are appropriately investigated and resolved.

## Conflict of Interest Statement

The authors declare that the research was conducted in the absence of any commercial or financial relationships that could be construed as a potential conflict of interest.

## References

[1] Ikram MK Cheung CY Wong TY Chen CP Retinal pathology as biomarker for cognitive impairment and Alzheimer’s disease. J Neurol Neurosurg Psychiatry (2012) 83:917–22. 10.1136/jnnp-2011-301628 22733082

[2] London A Benhar I Schwartz M. The retina as a window to the brain-from eye research to CNS disorders. Nat Rev Neurol (2013) 9:44–53. 10.1038/nrneurol.2012.227 23165340

[3] Cheung CY Ikram MK Chen C Wong TY Imaging retina to study dementia and stroke. Prog Retin Eye Res (2017) 57:89–107. 10.1016/j.preteyeres.2017.01.001 28057562

[4] Huang D Swanson EA Lin CP Schuman JS Stinson WG Chang W Optical coherence tomography. Science (1991) 254(5035):1178–81. 10.1126/science.1957169 1957169 PMC4638169

[5] Thomson KL Yeo JM Waddell B Cameron JR Pal S. A systematic review and meta-analysis of retinal nerve fiber layer change in dementia, using optical coherence tomography. Alzheimers Dement (Amst) (2015) 1(2):136–43. 10.1016/j.dadm.2015.03.001 27239501 PMC4876885

[6] Paquet C Boissonnot M Roger F Dighiero P Gil R Hugon J Abnormal retinal thickness in patients with mild cognitive impairment and Alzheimer’s disease. Neurosci Lett (2007) 420:97–9. 10.1016/j.neulet.2007.02.090 17543991

[7] Berisha F Feke GT Trempe CL McMeel JW Schepens CL Retinal abnormalities in early Alzheimer’s disease. Invest Ophthalmol Vis Sci (2007) 48:2285–9. 10.1167/iovs.06-1029 17460292

[8] Lu Y Li Z Zhang X Ming B Jia J Wang R Retinal nerve fiber layer structure abnormalities in early Alzheimer’s disease: evidence in optical coherence tomography. Neurosci Lett (2010) 480:69–72. 10.1016/j.neulet.2010.06.006 20609426

[9] Kesler A Vakhapova V Korczyn AD Naftaliev E Neudorfer M Retinal thickness in patients with mild cognitive impairment and Alzheimer’s disease. Clin Neurol Neurosurg (2011) 113:523–6. 10.1016/j.clineuro.2011.02.014 21454010

[10] Marziani E Pomati S Ramolfo P Cigada M Giani A Mariani C Evaluation of retinal nerve fiber layer and ganglion cell layer thickness in Alzheimer’s disease using spectral-domain optical coherence tomography. Invest Ophthalmol Vis Sci (2013) 54:5953–8. 10.1167/iovs.13-12046 23920375

[11] Cheung CY Ong YT Hilal S Ikram MK Low S Ong YL Retinal ganglion cell analysis using high-definition optical coherence tomography in patients with mild cognitive impairment and Alzheimer’s disease. J Alzheimers Dis (2015) 45(1):45–56. 10.3233/JAD-141659 25428254

[12] Kirbas S Turkyilmaz K Anlar O Tufekci A Durmus M. Retinal nerve fiber layer thickness in patients with Alzheimer disease. J Neuroophthalmol (2013) 33(1):58–61. 10.1097/WNO.0b013e318267fd5f 22918296

[13] Shi Z Zhu Y Wang M Wu Y Cao J Li C The utilization of retinal nerve fiber layer thickness to predict cognitive deterioration. J Alzheimers Dis (2015) 49(2):399–405. 10.3233/JAD-150438 26484909

[14] Jones-Odeh E Hammond CJ How strong is the relationship between glaucoma, the retinal nerve fibre layer, and neurodegenerative diseases such as Alzheimer’s disease and multiple sclerosis? Eye (Lond) (2015) 29(10):1270–84. 10.1038/eye.2015.158 26337943 PMC4815693

[15] Ong YT Hilal S Cheung CY Venketasubramanian N Niessen WJ Vrooman H Retinal neurodegeneration on optical coherence tomography and cerebral atrophy. Neurosci Lett (2015) 584:12–6. 10.1016/j.neulet.2014.10.010 25451722

[16] Coppola G Di Renzo A Ziccardi L Martelli F Fadda A Manni G Optical coherence tomography in Alzheimer’s disease: a meta-analysis. PLoS One (2015) 10(8):e0134750 10.1371/journal.pone.0134750 26252902 PMC4529274

[17] Koh VT Tham YC Cheung CY Wong WL Baskaran M Saw SM Determinants of ganglion cell-inner plexiform layer thickness measured by high-definition optical coherence tomography. Invest Ophthalmol Vis Sci (2012) 53:5853–9. 10.1167/iovs.12-10414 22836772

[18] Mwanza JC Oakley JD Budenz DL Chang RT Knight OJ Feuer WJ. Macular ganglion cell-inner plexiform layer: automated detection and thickness reproducibility with spectral domain-optical coherence tomography in glaucoma. Invest Ophthalmol Vis Sci (2011) 52:8323–9. 10.1167/iovs.11-7962 21917932 PMC3208140

[19] Francoz M Fenolland JR Giraud JM El Chehab H Sendon D May F Reproducibility of macular ganglion cell-inner plexiform layer thickness measurement with cirrus HD-OCT in normal, hypertensive and glaucomatous eyes. Br J Ophthalmol (2014) 98(3):322–8. 10.1136/bjophthalmol-2012-302242 24307717

[20] Kim KE Yoo BW Jeoung JW Park KH. Long-term reproducibility of macular ganglion cell analysis in clinically stable glaucoma patients. Invest Ophthalmol Vis Sci (2015) 56(8):4857–64. 10.1167/iovs.14-16350 25829417

[21] Carpineto P Aharrh-Gnama A Ciciarelli V Mastropasqua A Di Antonio L Toto L Reproducibility and repeatability of ganglion cell-inner plexiform layer thickness measurements in healthy subjects. Ophthalmologica (2014) 232(3):163–9. 10.1159/000362177 25115538

[22] Lee HJ Kim MS Jo YJ Kim JY Ganglion cell inner plexiform layer thickness in retinal diseases: repeatability study of spectral-domain optical coherence tomography. Am J Ophthalmol (2015) 160(2):283–9. 10.1016/j.ajo.2015.05.015 26004405

[23] Leung CK Cheung CY Weinreb RN Qiu Q Liu S Li H Retinal nerve fiber layer imaging with spectral-domain optical coherence tomography: a variability and diagnostic performance study. Ophthalmology (2009) 116(7):1257–63, 1263.e1–2. 10.1016/j.ophtha.2009.04.013 19464061

[24] Leung CK Cheung CY Lin D Pang CP Lam DS Weinreb RN Longitudinal variability of optic disc and retinal nerve fiber layer measurements. Invest Ophthalmol Vis Sci (2008) 49(11):4886–92. 10.1167/iovs.07-1187 18539940

[25] Wadhwani M Bali SJ Satyapal R Angmo D Sharma R Pandey V Test-retest variability of retinal nerve fiber layer thickness and macular ganglion cell-inner plexiform layer thickness measurements using spectral-domain optical coherence tomography. J Glaucoma (2015) 24(5):109–15. 10.1097/IJG.0000000000000203 25517254

[26] Polo V Garcia-Martin E Bambo MP Pinilla J Larrosa JM Satue M Reliability and validity of Cirrus and Spectralis optical coherence tomography for detecting retinal atrophy in Alzheimer’s disease. Eye (Lond) (2014) 28(6):680–90. 10.1038/eye.2014.51 24625377 PMC4058616

[27] Petersen RC Mild cognitive impairment as a diagnostic entity. J Intern Med (2004) 256(3):183–94. 10.1111/j.1365-2796.2004.01388.x 15324362

[28] Nouri-Mahdavi K Nowroozizadeh S Nassiri N Cirineo N Knipping S Giaconi J Macular ganglion cell/inner plexiform layer measurements by spectral domain optical coherence tomography for detection of early glaucoma and comparison to retinal nerve fiber layer measurements. Am J Ophthalmol (2013) 156(6):1297–307. 10.1016/j.ajo.2013.08.001 24075422 PMC3834195

[29] Hua Z Fang Q Sha X Yang R Hong Z. Role of retinal nerve fiber layer thickness and optic disk measurement by OCT on early diagnosis of glaucoma. Eye Sci (2015) 30(1):7–12. 26390791

[30] Leung CK Yu M Weinreb RN Ye C Liu S Lai G Retinal nerve fiber layer imaging with spectral-domain optical coherence tomography: a prospective analysis of age-related loss. Ophthalmology (2012) 119(4):731–7. 10.1016/j.ophtha.2011.10.010 22264886

[31] Leung CK Ye C Weinreb RN Yu M Lai G Lam DS. Impact of age-related change of retinal nerve fiber layer and macular thicknesses on evaluation of glaucoma progression. Ophthalmology (2013) 120(12):2485–92. 10.1016/j.ophtha.2013.07.021 23993360

