# Supplementary material for: Repeatability and Reproducibility of Retinal Neuronal and Axonal Measures on Spectral-Domain Optical Coherence Tomography in Patients with Cognitive Impairment
Source: Front Neurol. 2017 Aug 15;8:359. doi: 10.3389/fneur.2017.00359 (PMC5559462; doi:10.3389/fneur.2017.00359)
Supplement: Supplementary file 1 [file Data_Sheet_1.docx]

**Repeatability and Reproducibility of Retinal Neuronal and Axonal Measures on Spectral-domain Optical Coherence Tomography in Patients with Cognitive Impairment**

**Supplemental Materials**

**Appendix:**

Appendix 1: APOSTLE OCT Reporting Criteria


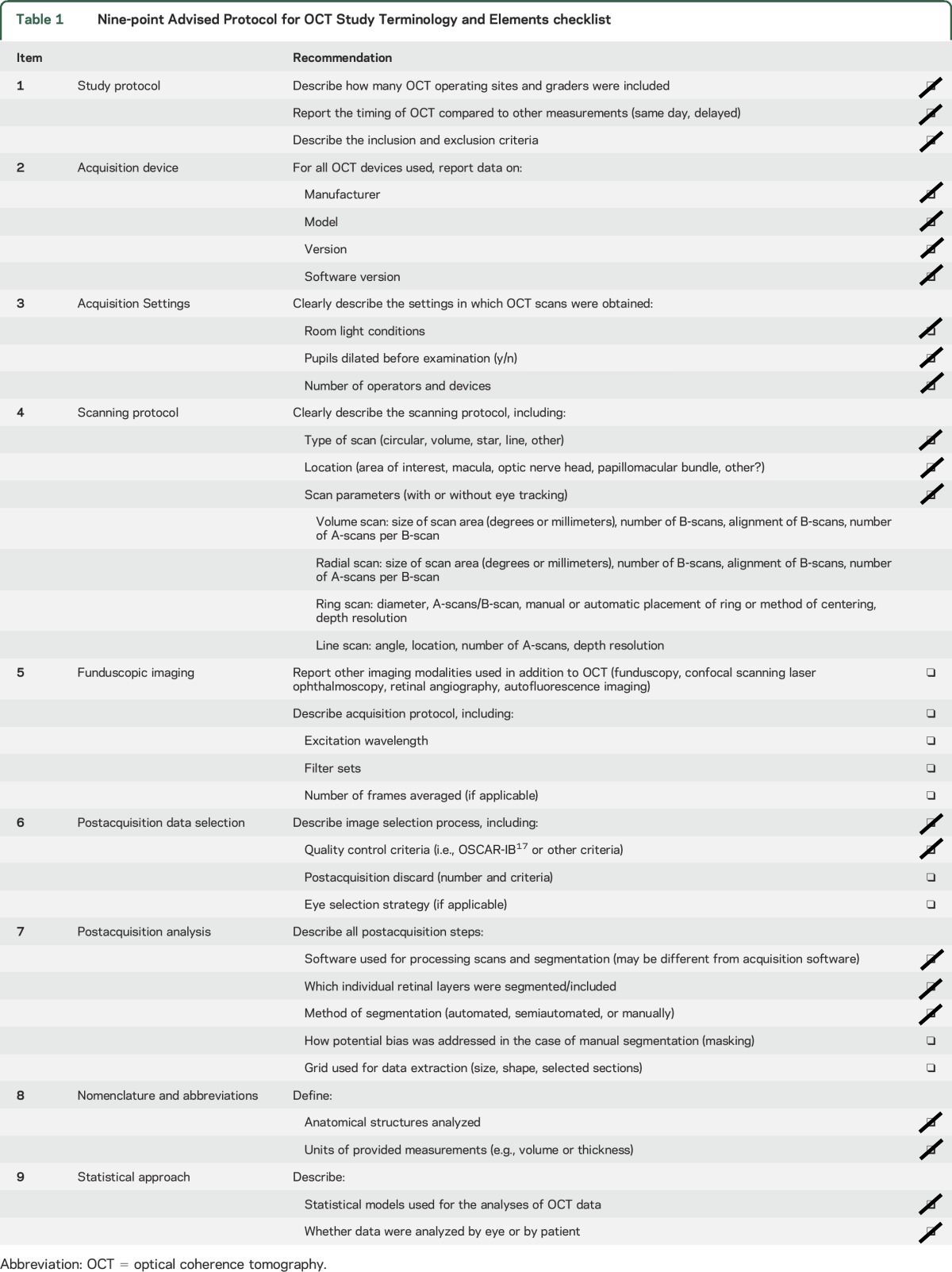


**Appendix 2**: Breakdown of included/excluded subject eyes for the inter-visit reproducibility assessment

| **Macular GC-IPL** | **Right Eye** | **Left Eye** |
| --- | --- | --- |
| Total eligible/ included: | 26 | 26 |
| Total excluded: | 35 | 35 |
| - Any visit with incomplete scan | 6 | 3 |
| - Retinal pathology | 7 | 7 |
| - Poor fixation | 3 | 5 |
| - Segmentation error | 19 | 20 |
|  |  |  |
| **ONH RNFL** | **Right Eye** | **Left Eye** |
| Total: eligible/ included: | 37 | 32 |
| Total excluded: | 24 | 29 |
| - Any visit with incomplete scan | 6 | 6 |
| - Retinal pathology | 0 | 1 |
| - Poor fixation | 6 | 5 |
| - Segmentation error | 12 | 17 |

**Appendix 3**: Breakdown of included/excluded subject eyes for the intra-visit reproducibility assessment

| **Macular GC-IPL** | **Right Eye** | **Left Eye** |
| --- | --- | --- |
| Total eligible/ included: | 37 | 33 |
| Total excluded: | 32 | 36 |
| - Retinal pathology | 8 | 8 |
| - Poor fixation | 4 | 4 |
| - Segmentation error | 20 | 24 |
|  |  |  |
| **ONH RNFL** | **Right Eye** | **Left Eye** |
| Total eligible/ included: | 35 | 42 |
| Total excluded: | 30 | 34 |
| - Retinal pathology | 7 | 6 |
| - Poor fixation | 6 | 5 |
| - Segmentation error | 17 | 23 |

**Supplemental Tables:**

**Supplemental Table 1:** Intra-visit repeatability and inter-visit reproducibility of macular ganglion cell-inner plexiform layer (GC-IPL) measurement in AD subjects.

| Parameters | CoV | ICC (95% CI) | |
| --- | --- | --- | --- |
| MAC Intra Visit Parameters (AD) n=37 | | | |
| GC-IPL Average Thickness | 1.06 | 0.967 | (0.936-0.983) |
| GC-IPL Minimum Thickness | 2.43 | 0.829 | (0.694-0.908) |
| GC-IPL Temporal Superior | 1.25 | 0.966 | (0.935-0.982) |
| GC-IPL Superior | 1.92 | 0.945 | (0.896-0.971) |
| GC-IPL Nasal Superior | 1.60 | 0.955 | (0.915-0.977) |
| GC-IPL Nasal Inferior | 2.09 | 0.920 | (0.852-0.958) |
| GC-IPL Inferior | 1.97 | 0.876 | (0.774-0.934) |
| GC-IPL Temporal Inferior | 1.34 | 0.954 | (0.912-0.976) |
| MAC Inter Visit Parameters (AD) n=24 | | | |
| GC-IPL Average Thickness | 1.12 | 0.965 | (0.932-0.983) |
| GC-IPL Minimum Thickness | 1.73 | 0.904 | (0.822-0.954) |
| GC-IPL Temporal Superior | 1.42 | 0.952 | (0.907-0.978) |
| GC-IPL Superior | 2.05 | 0.914 | (0.841-0.959) |
| GC-IPL Nasal Superior | 1.75 | 0.960 | (0.924-0.981) |
| GC-IPL Nasal Inferior | 2.28 | 0.918 | (0.858-0.961) |
| GC-IPL Inferior | 2.17 | 0.884 | (0.789-0.944) |
| GC-IPL Temporal Inferior | 1.93 | 0.887 | (0.785-0.947) |

**Supplemental Table 2.** Intra-visit repeatability and inter-visit reproducibility of macular ganglion cell-inner plexiform layer (GC-IPL) measurement in MCI subjects.

| Parameters | CoV | ICC (95% CI) | |
| --- | --- | --- | --- |
| MAC Intra Visit Parameters (MCI) n=33 | | | |
| GC-IPL Average Thickness | 1.26 | 0.991 | (0.982-0.996) |
| GC-IPL Minimum Thickness | 1.84 | 0.988 | (0.976-0.994) |
| GC-IPL Temporal Superior | 1.77 | 0.987 | (0.974-0.994) |
| GC-IPL Superior | 1.87 | 0.971 | (0.943-0.986) |
| GC-IPL Nasal Superior | 1.45 | 0.983 | (0.967-0.992) |
| GC-IPL Nasal Inferior | 2.63 | 0.959 | (0.918-0.979) |
| GC-IPL Inferior | 3.01 | 0.974 | (0.949-0.987) |
| GC-IPL Temporal Inferior | 1.87 | 0.990 | (0.979-0.995) |
| MAC Inter Visit Parameters (MCI) n=28 | | | |
| GC-IPL Average Thickness | 1.35 | 0.989 | (0.980-0.995) |
| GC-IPL Minimum Thickness | 1.84 | 0.991 | (0.984-0.996) |
| GC-IPL Temporal Superior | 2.43 | 0.962 | (0.931-0.981) |
| GC-IPL Superior | 2.56 | 0.949 | (0.908-0.974) |
| GC-IPL Nasal Superior | 1.73 | 0.984 | (0.971-0.992) |
| GC-IPL Nasal Inferior | 1.98 | 0.972 | (0.948-0.986) |
| GC-IPL Inferior | 2.32 | 0.980 | (0.964-0.990) |
| GC-IPL Temporal Inferior | 1.80 | 0.987 | (0.977-0.994) |

**Supplemental Table 3:** Intra-visit repeatability and inter-visit reproducibility of retinal nerve fiber layer (RNFL) measurement in AD subjects

| Parameters | CoV | ICC (95%CI) | |
| --- | --- | --- | --- |
| RNFL Intra Visit Parameters (AD) n=35 | | | |
| RNFL Average Thickness | 1.32 | 0.982 | (0.965-0.991) |
| Temporal Quadrant | 2.24 | 0.967 | (0.354-0.983) |
| Superior Quadrant | 2.33 | 0.965 | (0.933-0.982) |
| Nasal Quadrant | 3.17 | 0.942 | (0.888-0.970) |
| Inferior Quadrant | 2.67 | 0.971 | (0.944-0.985) |
| RNFL Inter Visit Parameters (AD) n=29 | | | |
| RNFL Average Thickness | 2.63 | 0.951 | (0.910-0.975) |
| Temporal Quadrant | 2.82 | 0.967 | (0.912-0.983) |
| Superior Quadrant | 3.79 | 0.917 | (0.854-0.957) |
| Nasal Quadrant | 5.29 | 0.890 | (0.809-0.943) |
| Inferior Quadrant | 3.68 | 0.951 | (0.911-0.975) |

**Supplemental Table 4:** Intra-visit repeatability and inter-visit reproducibility of retinal nerve fiber layer (RNFL) measurement in MCI subjects

| Parameters | CoV | ICC (95%CI) | |
| --- | --- | --- | --- |
| RNFL Intra Visit Parameters (MCI) n=42 | | | |
| RNFL Average Thickness | 1.35 | 0.987 | (0.974-0.993) |
| Temporal Quadrant | 2.73 | 0.930 | (0.873-0.962) |
| Superior Quadrant | 2.45 | 0.976 | (0.956-0.987) |
| Nasal Quadrant | 2.73 | 0.918 | (0.853-0.955) |
| Inferior Quadrant | 2.37 | 0.981 | (0.964-0.990) |
| RNFL Inter Visit Parameters (MCI) n=40 | | | |
| RNFL Average Thickness | 2.56 | 0.952 | (0.920-0.972) |
| Temporal Quadrant | 3.61 | 0.957 | (0.929-0.975) |
| Superior Quadrant | 3.90 | 0.926 | (0.878-0.957) |
| Nasal Quadrant | 5.01 | 0.790 | (0.677-0.874) |
| Inferior Quadrant | 3.84 | 0.957 | (0.929-0.975) |
